# Tropical almond antioxidant gels mitigate the adverse effects of strong whitening agents on immediate bonding

**DOI:** 10.1590/1807-3107bor-2025.vol39.072

**Published:** 2025-07-07

**Authors:** Verónica Cecilia MEJÍA, Vitória Moraes MARQUES, Tamara Gonçalves de ARAÚJO, Lara Cecília de MOURA, Ana Cristina de Mello FIALLOS, Mary Anne Sampaio de MELO, Sérgio Lima SANTIAGO, Vanara Florêncio PASSOS

**Affiliations:** (a) Universidade Federal do Ceará – UFC, Faculty of Pharmacy, Nursing and Dentistry, Department of Operative Dentistry, Fortaleza, CE, Brazil.; (b) Universidade Federal do Ceará – UFC, Faculty of Pharmacy, Nursing and Dentistry, Department of Pharmacy, Fortaleza, CE., Brazil.; (c) University of Maryland, School of Dentistry, Department of Comprehensive Dentistry, Baltimore, MD, EUA.

**Keywords:** Reactive Oxygen Species, Hydrogen Peroxide, Ascorbic Acid, Dental Cements, Dental Adhesives, Dental Enamel

## Abstract

Radicals from tooth whitening products can reduce bond strength, posing challenges for dentists when a bonding procedure must be performed in teeth immediately after whitening. This study aimed to evaluate the antioxidant activity (AA) of *Terminalia catappa* Linn (TCL) leaf extract as a potential agent to mitigate the negative impact of high-concentration whitening agents on immediate bonding performance. The AA of green and ripe leaf extracts was measured using the 2,2-di-phenyl-1-picrylhydrazyl radical scavenging capacity assay (DPPH). To determine the TCL influence on bond strength, six groups of bovine enamel blocks (n=10) were created. Group 1 was the positive control (unbleached enamel), while Group 2 was the negative control (no antioxidant treatment). Groups 3 to 6 were bleached and treated with 10% sodium ascorbate (SA) or 0.1%, 0.2%, and 0.3% TCL gels. Adhesion was conducted using a two-step conventional system and dental composite resin. Microtensile testing was performed after 24 hours, and data were analyzed by one-way ANOVA with Tukey’s post hoc test (p > 0.05). From the DPPH assay, results with IC50 < 50 µg/mL indicate high AA for all tested extracts. This method established a difference of around 12 times more AA for the TCL-hydroalcoholic extract of green leaf to the aqueous extract of ripe leaves. There were no significant differences in bond strength among groups treated with TCL-hydroalcoholic extract of green leaf gels (p > 0.05) and unbleached enamel. Tropical almond-derived antioxidant gels emerge as a promising strategy to enhance immediate bond strength on enamel after high-concentration in-office whitening treatments.

## Introduction

The increasing demand for rapid and noticeable tooth whitening and the challenge of whitening areas with deep staining, such as discoloration after root canal therapy, continue to drive the popularity of high-concentration hydrogen peroxide treatments.^1^

High-concentration hydrogen peroxide, often at a concentration of 35–38%, provides a potent solution capable of penetrating deeper into the tooth structure to achieve significant whitening effects where lower concentrations may fail.^2^ This demand stresses the difficult balance that patients and dental professionals navigate between achieving desired aesthetic outcomes and maintaining the tooth’s integrity.^2,3^

Tooth whitening aims to increase the light reflection of the teeth by modifying or removing pigment molecules in mineralized tissues. Oxygen free radicals (OFR), which include the superoxide anion (O _2_−·), the hydroxyl radical (H.O. •), and other oxygen species like H_2_O_2_ and singlet oxygen O_2_ (1Δg), are generated through the dissociation of hydrogen peroxide, which is present as an active ingredient either as the primary or secondary component (carbamide peroxide) in whitening products. OFRs are responsible for the chemical oxidation of these pigments.^4^ Despite being considered a simple and non-invasive procedure, tooth whitening can have secondary local effects on the oral mucosa, dental tissues, and restorative materials, such as gingival irritation, tooth hypersensitivity, micromorphological changes in enamel and dental restorations.^3,5^ The decrease in bond strength of adhesives applied to enamel/dentin in the same session as tooth whitening is one of the most prevalent and inevitable side effects.^6^

Changes such as increased surface porosity of the enamel and retention of OFRs and residual hydrogen peroxide have been associated with interference in the proper infiltration of adhesives into the tissues and adequate material polymerization.^7-9^ Studies have shown that this undesirable effect can be reversed, and the intensity of its occurrence varies according to the whitening agent used, including application time and concentration.^7,9^ Therefore, it is recommended to wait one to three weeks after completion of whitening before applying the adhesive protocol to preserve proper bond strength.^8,9^

Another approach to achieve immediate benefits in mitigating these side effects is using antioxidant agents, which aim to partially or fully neutralize the residual OFRs.^8,10^ Therefore, several studies have focused on these substances comparing their effects and protocols.^8,10-13^ Promising results have been obtained in in vitro experiments, demonstrating the benefits of certain phenolic compounds such as those found in green tea extract (catechins) and grape seeds (proanthocyanidins), as well as other agents such as alpha-tocopherol and sodium ascorbate (SA).^8,11,14^

However, the literature has highlighted some limitations of certain antioxidants.^8,11^ Sodium ascorbate, which is extensively researched, is characterized by high molecular instability, which can potentially lead to substrate staining. High concentrations or prolonged exposure to this agent may also impair adhesion by forming crystals in dentin or leaving residues on enamel. Regarding grape seed and pine bark extracts, their dark coloration raises concerns about potential impacts on the stability of the dental shade.

Exploring new natural antioxidants can lead to the development of more efficient application protocols and their adaptation to various clinical conditions, as certain antioxidants may perform better in different contexts.^8^ Similarly, searching for new natural sources helps improve accessibility, which can reduce production costs and increase benefits for the population. Moreover, it promotes sustainability by having an ethical commitment to the responsible use of resources.

*Terminalia Catappa* Linn (TCL) is a tree of the Combretaceae family, native to tropical Asia and is now distributed across coastal areas of tropical and subtropical regions. Studies have identified interesting biological properties of TCL, such as antioxidant, anti-inflammatory, antimicrobial, and antifungal actions. The antioxidant effect of the tree is attributed to its high concentration of phenolic compounds in the leaves, including tannins and polyphenols such as gallic acid, ellagic acid, and punicalagin.^15,16^

Research on TCL has focused on its antibacterial properties in the oral cavity.^17,18^ However, its antioxidant activity in the context of dental bleaching remains unexplored. This study aimed to investigate the antioxidant potential of extracts from the green and ripe leaves of TCL. Also, it evaluated the impact on the bond strength of bleached bovine enamel using hydroalcoholic gels derived from green leaf extracts at three different concentrations (0.01%, 0.2%, and 0.3%).

## Methods

### Experimental design

**Figure 1:**
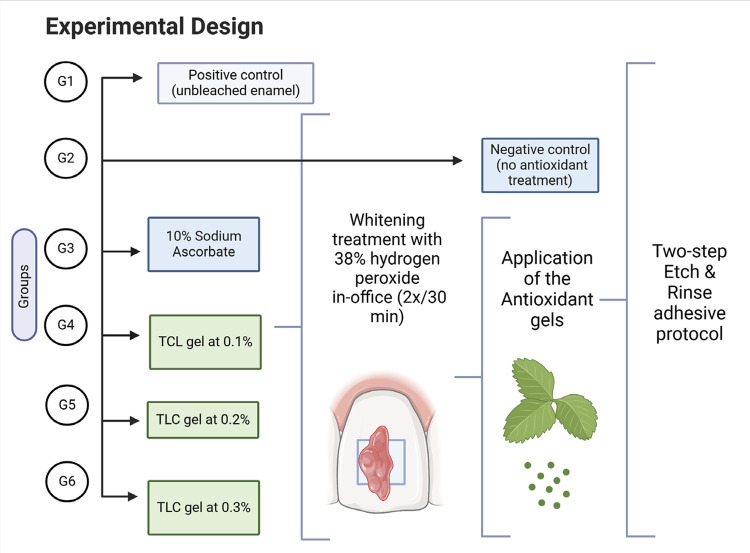
Treatment procedures and methodology in each study group. *TCL= Terminalia Catappa Linn.

The antioxidant activity of TCL leaf extracts was evaluated using the DPPH (2,2-diphenyl-1-picryl-hydrazyl-hydrate) method to determine the IC_50_, using vitamin C as the standard. To assess its impact on tissue bond strength after whitening, sixty bovine enamel blocks were randomly distributed into six experimental groups (n=10 each). In this study, the treatment protocol was the only evaluation factor, which was categorized into six levels: no dental whitening, whitening without antioxidant treatment, whitening with antioxidant treatment using 10% sodium ascorbate (S.A.) or hydroalcoholic TCL-gels at 0.1%, 0.2%, and 0.3%. The specimens were divided into the following groups: G1: positive control (unbleached enamel), G2 to G6 received whitening treatment with in-office whitening. G2: negative control (no antioxidant treatment). G3 to G6 were treated with the following antioxidant solutions: 10% sodium ascorbate (SA), 0.1%, 0.2%, and 0.3% TCL gels (Figure 1). After antioxidant treatment, all groups received a conventional two-step adhesive protocol on the same day, followed by increments of composite resin. The dependent variables analyzed were microtensile bond strength, expressed in MPa, and fracture pattern.

### TCL extracts and DPPH free radical assay

The extracts and antioxidant agents used in the study were prepared at the Department of Pharmacy, Faculty of Pharmacy, Dentistry, and Nursing of the Federal University of Ceará, Fortaleza , CE, Brazil. Green and ripe TCL leaves were collected on the campus under the exsiccata number 50917. Hydroalcoholic (70:30 water:ethanol) and aqueous extracts at 20% (drug:solvent) were prepared through maceration.

The methodology of Taghizadeh et al.,^19^ with modifications, was used to determine the free radical scavenging activity of DPPH. Vitamin C was used as the standard, and methanol was used as blank. First, 100 µL of the sample solvent and the standard were added to their respective wells, followed by serial dilution starting from 100 µg/mL up to 0.05 µ/mL. Then, 100 µL of a DPPH methanolic solution (80 µg/mL) was added to all wells except the blank, for negative control. After 30 min of incubation at room temperature protected from light, the absorbance was measured at 517 nm using a microplate reader (Synergy HT, Biotek, SP, Brazil).

**Figure 2:**
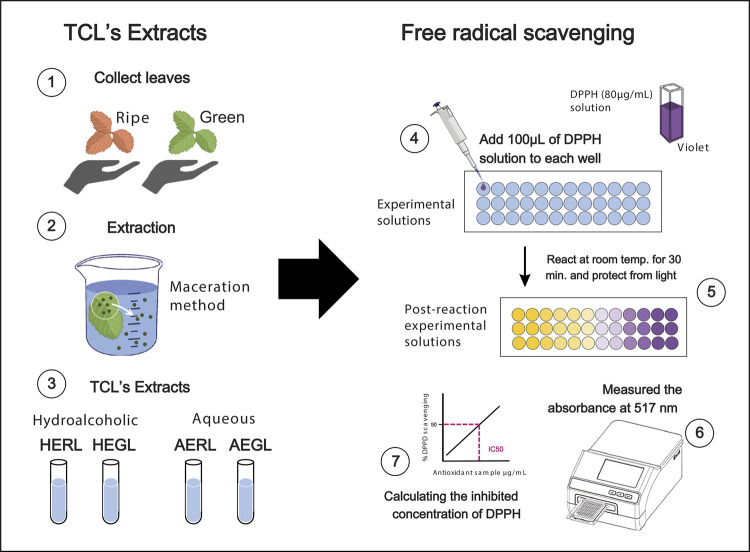
TCL extract preparation process and the DPPH free radical scavenging assay.

The IC_50_ (average inhibitory concentration) value was determined to indicate the amount of antioxidants required to neutralize 50% of the initial concentration of the DPPH radical. The following formula was used: % = (AC - AS) / AC × 100, where AC is the absorbance of the control and AS is the absorbance of the sample. This value is inversely proportional to the antioxidant capacity of the tested compound. Results with IC_50_ < 50 µg/mL indicate very strong antioxidant activity, values between 50 and 100 µg/mL are considered strong, 101 and 150 µg/mL moderate, 151 to 200 weak, while values greater than 200 µg/mL are considered inactive^20^. The process is illustrated in Figure 2.

*TCL: *Terminalia Catappa Linn*; HERL hydroalcoholic extract of ripe leaf; HEGL: hydroalcoholic extract of green leaf; AERL: aqueous extract of ripe leaf; AEGL: aqueous extract of green leaf. DPPH 2,2-diphenyl-1-picrylhydrazyl radical.

### Preparation of enamel bovine blocks

Bovine tooth crowns without fractures or fissures on the enamel surface were selected and stored in a 0.1% thymol solution. Enamel blocks (8x10 mm) were prepared on the buccal surface using a manual cutting machine (Isomet Low Speed Saw, Buehler Ltd., Lake Bluff, , U.S.A.) and a diamond cutting disc (Buehler Ltd., Joinville, Brazil) at low speed and under continuous cooling. The enamel surface was flattened with 180- and 600-grit Silicon carbide (SiC) wet sandpaper discs on a metallographic polisher (Arotec, Cotia, Brazil) at low speed and constant cooling. Blocks with dentin exposure after this procedure were excluded. Finally, the specimens were washed with distilled water in an ultrasonic cleaner (Ultracleaner 1400, Unique, Indaiatuba, Brazil) for five minutes and stored under refrigeration in sealed plastic containers, between gauze moistened with distilled water.

### Whitening procedure

A schedule was established to perform the procedures on two specimens from each group daily. Enamel blocks from groups 2 to 6 received two individual sessions of dental whitening, with a one-week interval, using 38% hydrogen peroxide (Potenza Bianco PRO SS 38% H_2_O_2_ On - P.H.S., Joinville, Brazil). Following the manufacturer’s instructions, the gel was applied on the dry surfaces for 30 min. After that, they were rinsed with profuse distilled water for 1 min, stored at 37°C, and immersed in artificial saliva.

### Preparation of antioxidant agents

Ten percent sodium ascorbate was obtained by dissolving 23 g of sodium ascorbate powder (Bottica Botanika, Louveira, Brazil) in 230 mL of distilled water, resulting in a solution with a pH of 5.7.

A dental gel formulation was developed containing hydroalcoholic extract of green TCL leaves at concentrations of 0.1%, 0.2%, and 0.3%, characterized by pH values of 5.05, 4.96, and 4.96, respectively. The base gel was produced using distilled water, glycerin, natrosol (Hidroxietilcelulose, Bottica Botanika, Louveira, Brazil), and phenoxyethanol; then, the concentrations were incorporated separately (Brazilian Filing Patent Number BR102018004126-6).

### Application of antioxidant agents

Immediately after the second whitening session, the antioxidants were applied for 10 min on the dry enamel surfaces, then rinsed with distilled water for 1 min. For G3, the specimens were constantly agitated and immersed in a plastic container with 8 mL of 10% SA. For G4, G5, and G6, each concentration of 0.1%, 0.2%, and 0.3% TCL gels, respectively, were manually applied on the surfaces using a micro-applicator (Cavibrush, FGM, Norte, Joinville, Brazil) with active circular movements.

### Bonding procedure and resin composite cover

A 37% phosphoric acid gel (Maquira, Maringá, Brazil) was applied on dry surfaces for 15 seconds and then rinsed with profuse distilled water for 30 seconds. The enamel was dried with absorbent paper; two drops of adhesive (Adapter Single Bond 2, 3M, Campinas, Brazil) were applied using a micro-applicator and gently agitated on the surface for 15 seconds. After removing the adhesive excess and evaporating the solvent using compressed air, the adhesive was light-cured (Valo Cordless Grand 3200 (Ultradent, Indaiatuba, Brazil) for 10 seconds. Then, two increments of Opallis composite resin (FGM, Joinville, Brazil), each with a thickness of 2 mm, were added and light-cured independently for 20 seconds. The specimens were immersed in artificial saliva in an incubator at 37ºC for 24 h.

### Microtensile bond strength test

After 24 h, the samples were cut at the mesio-distal and inciso-cervical directions using a diamond disk on a cutting machine at low speed, rotating at 200 rpm, under water cooling to obtain sticks with an area of approximately 1.0 ± 0.1 mm^2^.

Each stick was individually attached to a two-part free-sliding device using gel-based cyanoacrylate adhesive (Super Bonder gel, Loctite, Düsseldorf, Germany). The device was positioned in the universal testing machine (INSTRON 3345, São José dos Pinhais, Brazil), configured with a traction force at a 1 mm/min speed, using a load cell of 500 N (ISO/TS11405, 2003) until the specimens fractured. The adhesive strength was calculated as the ratio between the maximum force recorded during the test (N) and the bonding area (mm^2^), expressed in megapascals (MPa) and automatically provided by the equipment’s software.

### Fracture analysis

Each fractured specimen was analyzed by a single examiner using a stereoscopic microscope (Leica, Wetzlar, Germany) with a maximum magnification of 80x; the fracture pattern was identified according to the following criteria:

- Adhesive: fracture at the adhesive interface;
- Cohesive: fracture entirely within the enamel or resin composite;
- Mixed: fracture partially at the adhesive interface and partially cohesive within the enamel or resin.

### Statistical analysis

Kolmogorov-Smirnov test confirmed the normal distribution of the data in each group. The adhesive strength (MPa) between groups was compared with an analysis of variance test (one-way ANOVA), followed by a multiple comparison test with post hoc Tukey’s HSD (honestly significant difference). All analyses were performed using the Statistical Package for the Social Sciences (SPSS 22.0 for Windows, SPSS Inc., Chicago, USA.), adopting a significance level of 5%.

## Results

**Figure 3:**
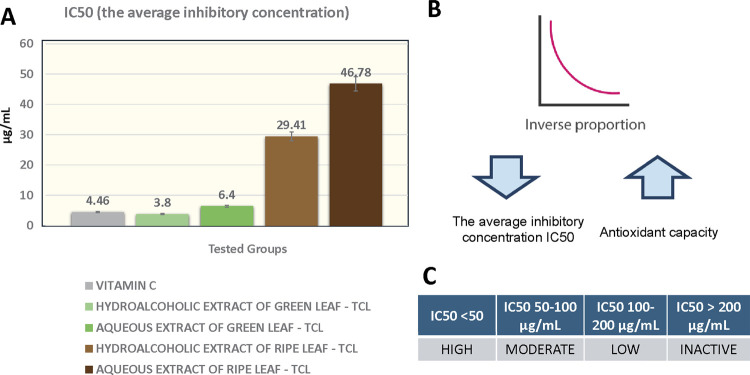
(A) Mean and standard deviation of the substances’ IC50s. (B) Illustration of the inverse correlation between IC50 values and antioxidant capacity of DPPH (2,2-diphenyl-1-picrylhydrazyl) radical inhibition. (C) Ranking of the antioxidative potential.

Mean values and standard deviations of the substances’ IC_50_s are presented in Figure 3. The lowest concentration to neutralize 50% of the initial concentration of the DPPH free radical (IC_50_) was obtained with the hydroalcoholic extract of TCL green leaf (3.80 ± 0.08), followed by vitamin C (4.46 ± 0.02) and the aqueous extract of green leaves (6.40 ± 0.05). Extracts from TCL mature leaves required a higher concentration to inhibit the free radical, with 29.41±0.01 for the hydroalcoholic extract and 46.78 ± 0.05 for the aqueous extract.

The adhesive bond strength in MPa for each group was calculated based on the adhesive and mix fracture microtensile results; the means and standard deviation were: G1 (positive control), 29.78 ± 12.4; G2 (negative control), 24.12 ± 9.77; G3 (10% SA), 28.26 ± 11.35; G4 (TCL-gel 0.1%), 26.03 ± 8.57; G5 (TCL-gel 0.2%), 25.94 ± 10.55; and G6 (TCL-gel 0.3%), 28.57 ± 10.74. The variance analysis between groups (one-way ANOVA) showed a statistically significant difference (p= 0.001). Among the antioxidant treatment groups, group G6 showed the highest values of resistance, followed by groups G3, G4, and G5.

The post-hoc multiple comparisons tests (Table) indicated a significant difference in adhesive bond strength of the group G2 (negative control) compared to Groups G1 (positive control) and G6 (0.3% TCL-gel) (p < 0.05).

**Table:** Mean (± SD) (MPa) for the different antioxidant treatments after whitening procedure.

Group		µTBS mean (±SD)
G1	Positive control	29.78 (12.44)^a^
G2	Negative control	24.12 (9.77)^b^
G3	Treatment with 10% SA	28.26 (11.35)^ab^
G4	Treatment with TCL 0.1%	26.03 (8.57)^ab^
G5	Treatment with TCL 0.2%	25.94 (10.55)^ab^
G6	Treatment with TCL 0.3%	28.57 (10.74)^a^

Means followed by distinct letters differ statistically at 5%, according to one-way ANOVA and Tukey’s test.

**Figure 4:**
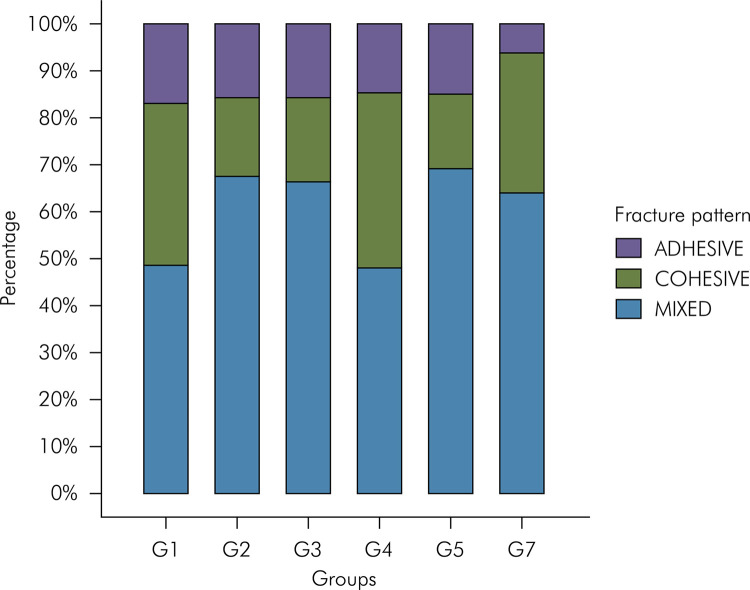
Analysis of fracture pattern for each experimental group. G1= positive control (unbleached enamel), G2= negative control (no antioxidant treatment), G3= 10% sodium ascorbate, G4= 0.1% *Terminalia Catappa Linn* (TCL) gel, G5= 0.2% TCL gel, G6= 0.3% TCL gel.

When analyzing the fracture patterns in each experimental group, mixed fractures predominated, followed by cohesive and adhesive fractures (Figure 4).

## Discussion

Research in alternative natural medicine has highlighted the phytotherapeutic potential of TCL, demonstrating its benefits for human health, including antimicrobial, anti-inflammatory, analgesic, wound healing, hepatoprotective, antioxidant, and other effects.^17,18,21,22^ The highest antioxidant potential has been demonstrated in leaf extracts, varying according to the maturation stage and extraction method. In this study, the antioxidant potential of leaves was evaluated at different stages of maturation, using both aqueous and hydroalcoholic extracts. The DPPH radical scavenging assay was chosen as the screening method due to its widespread use in plant extracts, ease of application, and high sensitivity.^19,23^

All TCL extracts showed IC_50_ values below 50 µg/mL, classifying them as highly active. The hydroalcoholic extract from green leaves exhibited a lower IC_50_ value than the standard used (Vit.C), indicating a higher antioxidant power. These findings are consistent with previous studies comparing TCL fruits at different stages of maturation^9^ with other species from the same genus, such as Terminalia fagifolia, *T. alata, T. bellirica*, and *T. corticosa*.^24^ Based on these results, the hydroalcoholic extract from green leaves was chosen to develop antioxidant gels at different concentrations.

Whitening products act by fragmenting and degrading chromatic macromolecules through reactive oxygen species.^4^ Residual oxygen in dental tissues has been associated with side effects in adhesion, resulting in inadequate infiltration and poor polymerization of monomers, affecting the quality and quantity of adhesive tags, as demonstrated in the classic study by Dishman et al.^25^ According to this evidence, the results in this study show lower mean values of bond strength in the group with whitening without antioxidant treatment (G2 - negative control). Previous studies have also reported this effect on the adhesive bond strength of enamel using different whitening concentrations and adhesive systems.^12,26,27^

Within the strategies used to eliminate residual oxygen, the utilization of antioxidant agents is especially appealing due to their ability to provide immediate outcomes.^8,11,14^ Several sources of antioxidants have been studied, among which sodium ascorbate has been the most investigated and compared in laboratory studies. Various protocols for the application of SA have been utilized, with a concentration of 10% solution and a duration of 10 minutes being the most extensively evaluated.^8,11^ In the present study, a 10% SA solution was applied with constant agitation for the same duration as described previously.

No statistically significant difference was observed in bond strength values between the group treated with SA (G3) and the positive control group (G1), the group bleached without treatment (G2), and the groups treated with TCL 0.1% and 0.2% (G4-G5). Our finding contrast with those of Silva et al.^13^ in 2011, who successfully restored adhesive strength by using the same SA protocol on enamel after whitening with 38% hydrogen peroxide. Murad et al.^28^ found that a 15-minute application of 10% SA gel achieved adhesive bond strength similar to that of the control group.

However, Ghaleb et al.^27^ discovered that applying SA using the drip method, with the same concentration and application time, improved adhesive strength to levels comparable to the control group, except for groups treated with 16% carbamide and 6% hydrogen peroxide. Studies suggest that these conflicting results are due to the significant influence of variables such as the concentration of whitening gels, the type of adhesive system, the number of antioxidant applications, and the consistency and duration of antioxidant application.^29,30^

The antioxidant activity of various plants and their derivatives is associated with the presence of phenolic compounds, such as flavonoids, tannins, lignin, and phenolic acids.^31^ Green tea extract contains abundant catechins, while grape seeds and pine bark contain proanthocyanidins, which have promoted their use in laboratory studies to evaluate improved adhesive strength in bleached tissues ^8,10^. Oyeniran et al.^32^ found a high concentration of phenolic compounds in the extract of TCL leaves, particularly ellagic and gallic acids, which can be attributed to the high antioxidant activity previously described by Fogaça et al.,^15^ Abdulkadir et al.,^23^ and Huang et al.^33^

Some studies have examined the antioxidant potential of TCL in dermatological products, demonstrating its protective effect against oxidative stress on skin cells.^22,33^ In dentistry, the antimicrobial properties of TCL leaf extract have been evidenced, reducing streptococcus mutans, Candida albicans, and Candida glabrata proliferation.^17,18^ Likewise, the recent research of Lobão et al.^18^ has demonstrated that after two years of water storage, primers with TCL maintained the adhesive-dentin interface properties while providing antimicrobial effects and promoting cross-linking with dentin.

In the present study, the data analysis showed the antioxidant effect of hydroalcoholic gels based on three different concentrations (0.1%, 0.2%, and 0.3%) of TCL leaf extracts to improve the bond strength on bovine enamel after bleaching with 38% hydrogen peroxide. The group that used the highest concentration (G6) showed no statistically significant difference compared to the control group. As evidenced by the literature review conducted by Barragué et al.,^11^ the antioxidant properties of natural sources can be a promising alternative to mitigate the adverse effects on dental bonding of composites after whitening.

This study represents the first step in exploring TCL’s antioxidant effects on dental bonding. The results indicate promising antioxidant properties of TCL and offer new avenues for its application in dentistry. However, it is important to acknowledge the limitations of this pioneering research. The clinically accepted waiting time of at least one week after dental whitening demonstrates consistent results in in vitro tests that improve adhesive strength. While the values only sometimes outperform those achieved with antioxidants,^8,10,18^ the absence of a systematic comparison with this alternative represents a gap in this research. El Zohairy et al.^34^ emphasized that in the microtensile test, forces are not applied directly to the adhesive interface of the samples, unlike the microshear test. Despite this distinction, it is crucial to note that both test modalities are suitable for this analysis.^34^ Another relevant aspect is that some studies have shown that the application of sodium ascorbate in hydrogel for periods exceeding 10 minutes can improve adhesive strength, with the additional advantage of more effective control during handling and application.^27,35^

It is important to consider the future clinical implications of implementing antioxidants to reverse the side effects of whitening on adhesion. Initially, this strategy involves additional steps to the clinical protocol, requiring compliance of the operator and dental team. On the other hand, substantial advantages can be highlighted, such as the potential optimization of service, allowing for more effective control of post-operative hypersensitivity attributed, according to some theories, to the diffusion of oxygen free radicals in the pulp chamber.^36,37^ Additionally, it is worth considering the advantage of providing a more efficient treatment plan in a single clinical session when using resin composites or in cases of bonding fixed appliances for orthodontic treatment.^2,10^

Further comprehensive studies are required to compare and discuss the results obtained with Terminalia extract in adhesive strength. They will broaden our understanding of its efficacy and safety, including cytotoxicity, microstructural evaluation of enamel and dentin, considering factors such as application times, comparison with different antioxidant agents, concentrations of whitening gels, and other adhesive systems.

## Conclusions

TCL extract is a novel and promising approach as a natural antioxidant agent for improving post-whitening adhesive bond strength. Further studies considering other variables are necessary to evaluate and compare the antioxidant effect of this extract.

## Data Availability

Data is available on demand from the reviewers.
